# A Case Series and Review of *Bacillus Cereus* Endocarditis from India

**DOI:** 10.2174/1874285801812010028

**Published:** 2018-03-30

**Authors:** Anusha Gopinathan, Anil Kumar, Amitabh C. Sen, Srisruthy Sudha, Praveen Varma, Sunil GS, Malini Eapen, Kavitha R. Dinesh

**Affiliations:** 1 Department of Microbiology, Amrita Institute of Medical Sciences, Amrita University, Clinical Assistant Professor, AIMS Ponekkara, Edappally, Kochi 682041, India; 2 Department of Microbiology, Amrita Institute of Medical Sciences, Amrita University, Clincial Professor, AIMS Ponekkara, Edappally, Kochi 682041, India; 3 Department of Anaesthesiology and Critical care medicine, Amrita Institute of Medical Sciences, Amrita University, Clinical Assistant Professor, AIMS Ponekkara, Edappally, Kochi 682041, India; 4 Department of Microbiology, Amrita Institute of Medical Sciences, Amrita University, Junior resident, AIMS Ponekkara, Edappally, Kochi 682041, India; 5 Department of Cardiovascular and Thoracic Surgery, Amrita Institute of Medical Sciences, Amrita University, Clinical professor and Head, AIMS Ponekkara, Edappally, Kochi 682041, India; 6 Department of Paediatric and Congenital heart surgery, Amrita Institute of Medical Sciences, Amrita University, Clinical professor, AIMS Ponekkara, Edappally, Kochi 682041, India; 7 Department of Pathology, Amrita Institute of Medical Sciences, Amrita University,, Clinical Professor, AIMS Ponekkara, Edappally, Kochi 682041, India; 8 Department of Microbiology, Amrita Institute of Medical Sciences, Amrita University, Clinical Professor, AIMS Ponekkara, Edappally, Kochi 682041, India

**Keywords:** Valvular heart disease, Endocarditis, Bacillus species, Gram positive bacilli, Bacteremia, Infant

## Abstract

**Introduction:**

Bacillus cereus is a gram positive bacilli found commonly in the soil and environment. It is a bacteria rarely associated with endocarditis.

**Case History:**

Intravenous drug abuse, presence of valvular defects, pacemakers, immunodeficiency are some of the known risk factors for *B.cereus* endocarditis. We present here a case series of two patients with *B.cereus* endocarditis along with a review of the literature.

**Conclusion:**

This is the first report of *B.cereus* endocarditis from India to the best of our knowledge.

## 1 INTRODUCTION

*Bacillus cereus* is an aerobic gram positive bacilli which is most often considered as a contaminant when isolated from blood culture. This bacteria can also cause deadly diseases in the presence of risk factors such as immunodeficiency history of drug abuse and pacemakers. The bacteria is known for its association with food poisoning, but it can also be responsible for infections such as osteomyelitis, respiratory tract infections, urinary tract infections, cutaneous infections, endophthalmitis, meningitis and rarely endocarditis [1]. Hence the isolation of *B.cereus* from blood culture must be given due importance in the presence of significant risk factors in the patient’s clinical history.

## 2 CASE HISTORY

### 2.1 Case 1

A five month old female baby was admitted for ventricular septal defect (VSD) repair for which she underwent VSD closure with a polytetrafluoroethylene (PTFE) (Gore-tex®) patch. On post operative day two, the baby developed persistent diarrhoea with occasional spikes of fever. Her clinical investigations showed normal haemoglobin(12.6g/dl) total white cell count (14.7K/µl) and erythrocyte sedimentation rate (12 mm/hr) but raised C- reactive protein(CRP)(121.2mg/dl). Sepsis was suspected and blood culture done in pediatric BacTAlert bottle (bioMerieux) yielded gram positive bacilli with central spores which was initially considered as a contaminant.

Two subsequent blood cultures also yielded the same Gram positive bacilli with central spores. The Gram positive bacilli was identified as *B. cereus* based on morphology on bacterial culture plates and standard biochemical tests. Antimicrobial susceptibility of the isolate was performed using Kirby Bauer disc diffusion testing according to CLSI guidelines [2] and were interpreted using breakpoints for *Staphylococcus aureus* . The isolate was found to be sensitive to vancomycin, amikacin, teicoplanin, piperacillin/tazobactam, cefoperazone/sulbactam, clindamycin and linezolid. It was resistant to penicillin G and trimethoprim-sulfamethoxazole. The identification of the isolate was confirmed by sequencing the 16SrRNA gene using universal primers [3].

Echocardiography (ECHO) performed on postoperative day four revealed an intact VSD patch with a thin strand like freely mobile hyper-echoic structure attached to the atrial tricuspid leaflet and choroid papillary muscle junction. An additional tiny apical muscular ventricular septal defect was also seen. Patient was thus diagnosed to have infective endocarditis and managed with intravenous vancomycin for ten days and continued with oral linezolid(40mg thrice daily) for 4 weeks. Patient improved with therapy and a repeat ECHO performed at the time of discharge and two weeks after discharge showed no vegetations. The patient continues to remain healthy and asymptomatic at 18 months follow-up.

### 2.2 Case 2

A 19 year old male presented with high grade fever for the past two weeks. He had been diagnosed with urinary tract infection and was on antibiotic therapy for the past one week with no improvement of symptoms. On clinical examination, the patient had a pansystolic murmur on the mitral area. All other system examinations were within normal limits. His lab investigations revealed normal hemoglobin(10.9g/dl), total white cell count(8400 K/µl) and platelet count(198 K/µl) with raised ESR(59 mm/hr), procalcitonin(0.392 ng/ml) and CRP(32.7mg/l). Paired aerobic blood cultures done in BactTAlert bottles were sterile.

Transoesophageal ECHO showed that the patient had an atrial myxoma measuring 2.16 x 0.8 cm probably arising from the lower part of the inter-atrial septum. The mass was protruding into the mitral valve causing severe eccentric mitral regurgitation. The patient was operated and the mass excised and sent for culture and histopathology. Histopathological examination of the vegetation revealed presence of amorphous eosinophilic material with nodular collections of basophilic round and rod like structures resembling bacterial colonies using Hematoxylin and Eosin stain and nodular colonies of Gram positive cocci and bacilli using Gram stain Figs. (**1** and **2**).

The excised mass on culture grew a gram positive bacilli which was identified as *B.cereus* using colony morphology and standard biochemical tests. The isolate was sensitive to gentamicin, vancomycin, tigecycline, and teicoplanin. It was resistant to penicillin G and trimethoprim -sulfamethoxazole. The bacteria was confirmed as *B.cereus* by matrix-assisted laser desorption ionization time-of-flight mass spectrometry (MALDI-TOF MS) (Bruker Daltonics, Bremen, Germany).

Post-operative period was uneventful. He was started on intravenous vancomycin 1 gm twice daily for 6 weeks. He responded to therapy and at the time of discharge patient had no vegetations on ECHO and subsequent blood cultures were also negative. He continues to remain asymptomatic without any evidence of recurrence at five months of follow-up.

## 3 DISCUSSION

*Bacillus cereus* is a a facultative anaerobic, spore forming gram positive bacilli which is found in soil, environment and hospital surroundings. The bacteria when cultured on 5% sheep blood agar produces dull opaque colonies with swarming and beta hemolysis [4]. It produces tissue destructive exoenzymes which are responsible for its pathogenicity. The bacteria is known to cause food poisoning and endophthalmitis. They can rarely cause severe infections such as bacterial pneumonia, brain abscess, meningitis, osteomyelitis, ocular keratitis, necrotizing skin and soft tissue infection . It is known to cause nosocomial acquired bacteremia and wound infections in postsurgical patients with intravascular devices such as catheters ^[^1^]^. *B.cereus* isolated from blood culture is most often considered as a contaminant [5]. According to Centres for Disease Control and Prevention(CDC), common skin contaminants such as *B.cereus* when isolated from blood can be considered as a pathogen if the patient exhibits signs and symptoms of sepsis and two or more blood culture drawn on separate occasions yield the same bacteria [6].

*B.cereus* is rarely associated with endocarditis. A review of cases reported in literature clearly shows that *B.cereus* endocarditis is most often seen in patients with history of intravenous drug abuse, central venous catheters, prosthetic heart valves, valvular heart disease, malignancy and immunosuppression. There is one case report of *B.cereus* endocarditis in a pregnant female with history of intravenous drug abuse [7].

The first case of *B.cereus* endocarditis was reported in a heroin addict in 1974. Since then there have been 24 reports of *B.cereus* endocarditis Table (**1**) [8-27].

ALL, acute lymphoblastic leukemia; ASD, atrial septal defect; AV, aortic valve; DA, drug abuse; IS, immunosuppression; Ins, instrumentation;MP-CVC, medical port central venous catheter; MV, mitral valve; Preg, Pregnant; PM, pacemaker; PV, prosthetic valve; TV, tricuspid valve; RA, right atrium; RHD, rheumatic heart disease; SVC, superior vena cava; VHD, valvular heart disease; VSD, ventral septal defect

Amk, amikacin; Cfz, cefazolin; Chl, chloramphenicol; Cip, ciprofloxacin; Cli, clindamycin; Cro, ceftriaxone; Cxm, cefuroxime; Ery, erythromycin; Gen, gentamicin; Kan, kanamycin; Lm, lincomycin; Mero, meropenem; Min, minocycline; Naf, nafcillin;Ofx, ofloxacin; Pen, penicillin; Pip, piperacillin; Rif, rifampicin; Str, streptomycin; Sxt, trimethoprim sulfamethoxazole; Tob, tobramycin;Van, vancomycin.

*B.cereus* endocarditis affects males more than females and is seen most often among the age group 41-60 years. Among the cardiac valves, mitral valve is more commonly involved followed by aortic and tricuspid valves. *B.cereus* endocarditis in majority of cases occurs after an episode of bacteremia, 88% of patients with endocarditis present with fever. Cutaneous colonisation, contamination of drug agents and injection equipment are the causative agents of bacteremia [4]. Kato **et al**. report 20% mortality among patients with *B.cereus* BSI [28]. Mortality rate of *B.cereus* bacteremia is higher in neonates compared to adults [29].Patients with prosthetic valve experience higher morbidity and mortality compared to those with native valve [22].

In most of the previous studies, prosthetic valve associated *B.cereus* endocarditis were treated using a combination of surgery and antimicrobial therapy and native valve endocarditis were treated with antimicrobial therapy alone. In our study, we report one native valve endocarditis which was treated with surgery and antimicrobial therapy. Surgery was necessitated due to the large size of the mass which was initially diagnosed as an atrial myxoma. Oh **et al*,* also report a case of native mitral valve endocarditis caused by *B.cereus* where surgery and antibiotics were used for therapy [24].

Antimicrobial therapy for *B.cereus* endocarditis is given for a period for 6 weeks. Monotherapy as well as combined therapy depending on the antimicrobial susceptibility of the isolate can be used. Though the bacteria is generally sensitive to vancomycin, aminoglycosides, imipenem, tigecycline, ciprofloxacin, chloramphenicol and linezolid, vancomycin appears to be the drug of choice [1, 4].The bacteria secretes beta-lactamase enzyme which is responsible for its resistance against penicillin and cephalosporins [1].

## CONCLUSION

This study underscores the significance of clinical correlation of *B.cereus* isolation from specimen cultures before reporting them as contaminants. *B.cereus*, which was so far believed to be hospital and environmental flora have now emerged as pathogens which can cause invasive disease.

## Figures and Tables

**Fig. (1):**
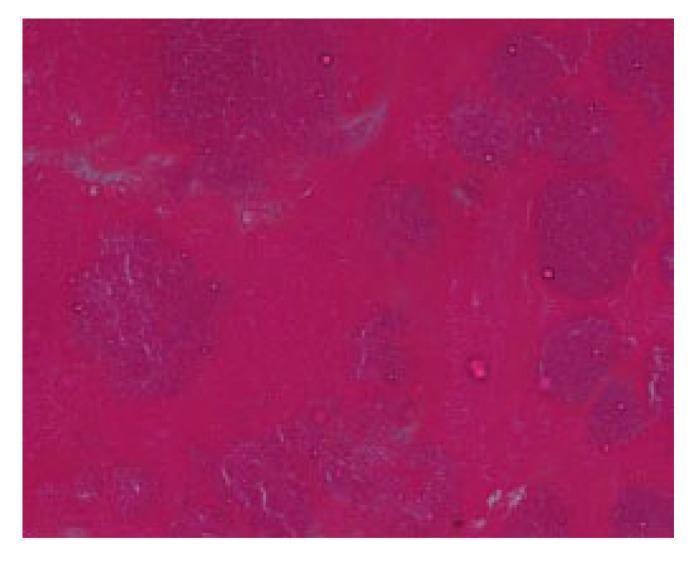
Histopathological examination of the vegetation (Hematoxylin and Eosin stain) showing amorphous eosinophilic material with nodular collections of basophilic round and rod like structures resembling bacterial colonies.

**Fig. (2):**
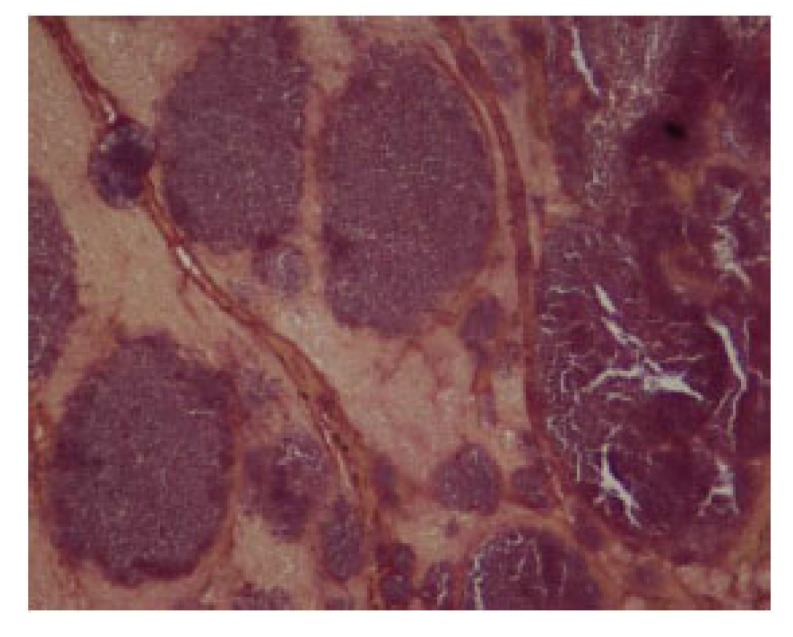
Histopathological examination of the vegetation (Gram Stain) showing nodular colonies of Gram positive cocci and bacilli.

**Table 1:** Summary of cases of bacillus cereus endocarditis ^[^4, 7-27^]^.

**S.No.**	**Author**	**Place**	**Age/** **Gender**	**Predisposing Factor**	**Valve**	**Treatment**	**Surgery**	**Clinical Outcome**
1	Craig **et al** 1974 ^[^8^]^	USA	18/F	ASD/DA	TV	Cli/Lm (5 weeks)	No	Recovered
2	Block **et al** 1978 ^[^9^]^	SA	51/F	PV	MV	Tob/Chl(NR)	No	Died
3	Tuazon**et al** 1979 ^[^10^]^	USA	NR	DA	NR	Naf(NR)	NR	Recovered
4	Tuazon**et al** 1979 ^[^10^]^	USA	NR	DA	NR	Cli	NR	Recovered
5	Tuazon**et al** 1979 ^[^10^]^	USA	NR	DA	NR	Chl, Ery,Gen (NR)	NR	Recovered
6	Wanvarie **et al** 1979 ^[^11^]^	Thailand	NR	RHD	AV	Pen, Gen, Str(NR)	No	Died
7	Weller **et al** 1979 ^[^12^]^	USA	50/F	DA	None	Cli/Kan(4 weeks)	No	Recovered
8	Oster **et al** 1982 ^[^13^]^	USA	55/M	PV	AV	Cli/Gen (6 weeks)	Yes	Recovered
9	Sliman**et al** 1987 ^[^14^]^	USA	43/F	PM, RHD	PM Wire	Cli(6 weeks)	Yes	Recovered
10	Steen **et al** 1992 ^[^15^]^	USA	34/M	PV	AV	Van(6 weeks)	Yes	Recovered
11	Tomomasa**et al** 1993 ^[^16^]^	Japan	12 month/F	IS, Ins	MV	NR	No	Recovered
12	Yamamura **et al** 1994 ^[^17^]^	Japan	43/M	PV	MV	Amk,Min(NR)	Yes	Recovered
13	Martin **et al** 1998 ^[^18^]^	Spain	NR	PV	MV	Gen, Rif,Van(NR)	Yes	Recovered
14	Castedo**et al** 1999 ^[^19^]^	Spain	45/F	PV	MV	Gen, Rif,Van(6 weeks)	Yes	Recovered
15	Cone LA **et al** 2005 ^[^20^]^	USA	38/M	ALL	MV	Pen, Van(3 weeks)	No	Died
16	Abusin S **et al** 2008 ^[^21^]^	USA	69/F	PM	Wire	Cfz(6 weeks)	No	Recovered
17	Thomas **et al** 2012 ^[^22^]^	USA	42/M	None	AV	Cro(6 weeks)	Yes	Recovered
18	Barraud O **et al** 2012 ^[^23^]^	France	65/M	PM	Wire	Ofx, Pip(4 weeks)	Yes	Recovered
19	Oh DH **et al** 2012 ^[^24^]^	Korea	54/M	VHD	MV	NR(6 weeks)	Yes	Recovered
20	Sharma **et al** 2013 ^[^25^]^	USA	5month/F	ALL	Junction of SVC and RA	Van, Mero(6 weeks)	No	Recovered
21	Ngow HA **et al** 2013^26]^	Malaysia	31/M	DA	AV	Cxm(6 weeks)	No	Recovered
22	Kitazawa **et al** 2015 ^[^27^]^	Japan	66/M	None	AV	Van(9 weeks)	Yes	Recovered
23	Shah M **et al** 2015 ^[^7^]^	USA	30/F	DA, Preg	TV	Van(6 weeks)	No	Recovered
24	Wright **et al** 2016 ^[^4^]^	USA	27/M	MP-CVC	TV	Van(6 weeks)	Yes	Recovered
25	Present study	India	5 month/F	VSD	TV	Van, Lin (4 weeks)	No	Recovered
26	Present study	India	19/M	Nil	MV	Van (6 weeks)	Yes	Recovered

NR, not reported ; F, Female; M, Male.
